# Early Molecular Testing for Presumptive Genetic Eye Diseases

**DOI:** 10.3390/genes17091092

**Published:** 2026-09-10

**Authors:** Marisol Ibarra-Ramírez, Jibran Mohamed-Noriega, Joel Arenas-Estala, David Asael Rodríguez-Torres, Luis Daniel Campos-Acevedo, Marissa L. Fernández-de-Luna

**Affiliations:** 1 Department of Genetics, University Hospital and Faculty of Medicine, Autonomous University of Nuevo León (UANL), Monterrey 64460, Mexico; mibarrar@uanl.edu.mx (M.I.-R.); joel.arenase@uanl.edu.mx (J.A.-E.); david.rodriguez@genetica-uanl.mx (D.A.R.-T.); lcamposa@uanl.edu.mx (L.D.C.-A.); 2 Department of Ophthalmology, University Hospital and Faculty of Medicine, Autonomous University of Nuevo León (UANL), Monterrey 64460, Mexico; jibran.mohamednrg@uanl.edu.mx

**Keywords:** genetic eye diseases, ophthalmic genetics, multigene panel, next-generation sequencing, molecular diagnosis, precision medicine

## Abstract

**Background**: Genetic eye diseases (GEDs) comprise a heterogeneous group of hereditary and de novo disorders that involve all ocular structures, including the retina, optic nerve, vitreous, anterior segment, and ocular development. Their marked clinical and genetic heterogeneity frequently delays diagnosis and access to genetic counseling, multidisciplinary management, and emerging gene-based therapies. Although ophthalmic deep phenotyping remains essential, we propose an approach that prioritizes genetic analysis, particularly in under-resourced healthcare settings with limited access to advanced imaging and electrophysiological tests. **Methods:** We conducted a retrospective observational study of 101 consecutive patients with suspected genetic eye diseases. All were evaluated at a tertiary referral center in northeastern Mexico between January 2020 and August 2023 through comprehensive ophthalmologic examination supplemented by optical coherence tomography, visual-field testing, and fundus photography when clinically indicated. All patients were offered early molecular testing while awaiting future tests, such as electrophysiological testing or further referral visits. Molecular testing consisted of an ophthalmology-focused next-generation sequencing multigene panel. **Results:** The primary outcome was molecular diagnostic yield. A clinically actionable molecular diagnosis was established in 45 patients, corresponding to an overall molecular diagnostic yield of 44.6%. A total of 165 variants were identified, including 70 pathogenic, 16 likely pathogenic, 43 variants of uncertain significance, and 36 likely benign variants. Usher syndrome was the most frequent molecular diagnosis (10/45, 22.2%), comprising nine patients with *USH2A*-associated Usher syndrome type 2 and one patient with *MYO7A*-associated Usher syndrome type 1B, followed by Stickler syndrome (8/45, 17.8%), oculocutaneous albinism (3/45, 6.7%), and Alström syndrome (2/45, 4.4%). Autosomal recessive disorders represented the predominant inheritance pattern (68.9%). **Conclusions:** Our findings support ophthalmology-focused multigene panels as an effective first-line diagnostic strategy for patients with suspected genetic eye diseases. Early molecular testing may shorten the diagnostic odyssey, optimize resource utilization, facilitate precision diagnosis and genetic counseling, and guide future targeted deep phenotypic evaluation, prognosis, and eligibility for emerging gene-specific therapies.

## 1. Introduction

Genetic eye diseases (GEDs) comprise a broad and highly heterogeneous group of disorders affecting all ocular structures, including the retina, optic nerve, vitreous, lens, anterior segment, and ocular development. Although individually rare, these conditions collectively represent a major cause of childhood blindness and irreversible visual impairment in young adults. Their clinical burden extends beyond vision loss because many GEDs are syndromic and may involve hearing impairment, neurological disease, renal abnormalities, skeletal manifestations, or multisystem dysfunction. An accurate molecular diagnosis is therefore essential for comprehensive management and genetic counseling [1,2,3,4].

Advances in genomic medicine have substantially expanded our understanding of the molecular basis of ocular disorders. Hundreds of genes have been implicated in GEDs, encompassing all Mendelian inheritance patterns as well as de novo pathogenic variants. This genetic heterogeneity is accompanied by phenotypic overlap, incomplete penetrance, and variable expressivity, which often complicate purely clinical diagnosis. Conversely, pathogenic variants in the same gene may produce distinct phenotypes, whereas clinically similar disorders may result from variants in different genes. These complexities have shifted ophthalmic genetics from an exclusively phenotype-driven discipline toward an integrated genotype–phenotype approach in which molecular testing plays a central diagnostic role [2,5,6,7,8].

Inherited retinal dystrophies (IRDs) constitute the largest and most extensively studied subgroup of GEDs. However, ophthalmology clinics evaluate a broader spectrum of conditions, including inherited optic neuropathies, vitreoretinal disorders, congenital ocular malformations, developmental anomalies, albinism, anterior segment dysgenesis, and syndromic ciliopathies. Restricting testing to narrow disease-specific panels based solely on the initial clinical impression may therefore miss atypical presentations and disorders with overlapping phenotypes. Comprehensive ophthalmology-focused multigene panels provide simultaneous assessment of genes associated with diverse ocular phenotypes while retaining high analytical sensitivity and practical clinical applicability [5,9,10,11,12,13,14,15].

The widespread implementation of next-generation sequencing (NGS) has transformed the diagnostic approach to GEDs. Targeted multigene panels offer strong diagnostic performance, lower cost, shorter turnaround times, and more focused interpretation than broader genomic approaches, making them suitable first-line tests in routine practice. Studies from Europe and Asia have reported molecular diagnostic yields of approximately 40–70%, depending on cohort composition, pre-test phenotyping, and sequencing strategy [9,10,11,12,13,14,15]. Mexican cohorts have similarly demonstrated the clinical value of NGS while revealing population-specific variant spectra and expanding the molecular landscape of ophthalmic genetic disorders in Latin America [16,17,18].

Traditional diagnostic pathways often emphasize extensive phenotypic characterization before molecular testing, including multimodal retinal imaging, optical coherence tomography (OCT), fundus autofluorescence, visual field testing, and visual electrophysiology [19,20,21]. These investigations provide essential structural and functional information; however, access to advanced imaging and standardized electrophysiological testing remains limited in many under-resourced healthcare systems. Requiring completion of every specialized study before genetic testing may prolong the diagnostic odyssey, delay genetic counseling and multidisciplinary evaluation, and postpone identification of patients who may benefit from gene-specific therapies.

A comparable evolution has occurred in inherited neuromuscular disorders, where NGS-based testing has increasingly moved earlier in the diagnostic pathway and may reduce reliance on invasive muscle biopsy or extensive electrophysiological studies before molecular confirmation. Functional and pathological investigations are now more often used selectively in unresolved cases or to refine disease severity, prognosis, and genotype–phenotype correlations [22,23,24]. We hypothesized that a similar pragmatic paradigm could be applicable to GEDs, particularly in healthcare settings with limited access to advanced electrophysiological investigations and repeated specialist visits.

Therefore, this study aimed to evaluate the clinical utility and molecular diagnostic yield of an ophthalmology-focused multigene panel used as a first-line diagnostic strategy in consecutive patients with suspected genetic eye diseases evaluated in routine practice at a tertiary referral center in northeastern Mexico. We also sought to characterize the molecular spectrum of disease in this population.

## 2. Materials and Methods

### 2.1. Study Design and Patient Population

This retrospective observational study was conducted at the Department of Medical Genetics in collaboration with the Department of Ophthalmology at the University Hospital, Universidad Autónoma de Nuevo León, Mexico. The study protocol was approved by the institutional Research and Ethics Committee and was performed in accordance with the Declaration of Helsinki and national regulations governing research involving human participants (Approval No. GN23-00001).

Consecutive patients referred from the Department of Ophthalmology between January 2020 and August 2023 with clinical suspicion of a genetic eye disease were considered eligible for inclusion. Patients of any age or sex were included provided they completed both the ophthalmologic evaluation and molecular genetic testing using the institutional ophthalmology-focused multigene panel. Patients with incomplete clinical information or without molecular testing were excluded from the analysis.

The study was designed to reflect routine clinical practice in a tertiary referral center, where patients are evaluated based on clinical suspicion and broad disease categories. Consequently, the cohort included individuals with a broad spectrum of ocular phenotypes involving retinal, optic nerve, vitreoretinal, anterior segment, developmental, and syndromic disorders.

### 2.2. Clinical Evaluation

All patients underwent a comprehensive ophthalmologic assessment performed by ophthalmologists experienced in the evaluation of genetic eye diseases. Clinical evaluation included a detailed personal and family history, pedigree construction when appropriate, best-corrected visual acuity, slit-lamp biomicroscopy, dilated fundus examination, and additional ophthalmic investigations according to clinical indication and local availability.

Complementary ophthalmic investigations were performed according to clinical indication and local availability and included optical coherence tomography (OCT), fundus autofluorescence (FAF), visual field testing, and fundus photography. Full-field electroretinography, when available, was performed in accordance with current International Society for Clinical Electrophysiology of Vision (ISCEV) standards [21]. Electrophysiological testing was rarely completed before panel testing and was generally reserved for unresolved cases, patients with variants of uncertain significance, or situations requiring additional functional characterization. These examinations were not mandatory for inclusion, reflecting the real-world setting in which access to specialized ophthalmic testing is limited.

Following ophthalmologic evaluation, all patients were referred to the Department of Medical Genetics, where clinical findings, family history, inheritance patterns, and available ancillary studies were integrated to determine the indication for molecular testing and to perform genotype–phenotype correlation after sequencing.

### 2.3. Molecular Genetic Analysis

Peripheral blood samples were obtained after written informed consent, and genomic DNA was extracted using standard laboratory procedures.

All patients underwent next-generation sequencing using a commercially available ophthalmology-focused multigene panel specifically designed for the molecular diagnosis of genetic eye diseases. The panel included more than 300 genes associated with inherited retinal disorders, optic neuropathies, vitreoretinal diseases, anterior segment dysgenesis, ocular developmental anomalies, albinism, ciliopathies, syndromic ophthalmic disorders, and other hereditary ocular conditions (Supplementary Table S1).

Target enrichment was performed using hybridization-based capture followed by paired-end sequencing on an Illumina platform. The assay achieved an average depth of coverage of at least 50× across targeted regions and was designed to detect single-nucleotide variants and small insertions/deletions in coding exons and flanking intronic regions. Copy-number variants, including exon-level deletions and duplications, were assessed using validated read-depth algorithms incorporated into the bioinformatic pipeline [25].

Sequence reads were aligned to the GRCh37/hg19 human reference genome. Population allele frequencies were assessed using gnomAD, ExAC, dbSNP, and disease-specific databases, including ClinVar and the Human Gene Mutation Database (HGMD), together with available peer-reviewed literature to support variant interpretation.

An ophthalmology-focused multigene panel was selected as the primary molecular diagnostic strategy because it provides comprehensive coverage of genes currently associated with genetic eye diseases while offering lower cost, simplified interpretation, and shorter turnaround time compared with broader genomic approaches. This strategy reflects routine clinical practice in our institution and was considered particularly suitable for evaluating its implementation as a first-line diagnostic test in an under-resourced healthcare setting.

### 2.4. Variant Interpretation

All identified sequence variants were interpreted according to the joint recommendations of the American College of Medical Genetics and Genomics and the Association for Molecular Pathology (ACMG/AMP) [26]. Variants were classified as pathogenic, likely pathogenic, of uncertain significance, likely benign, or benign.

Only pathogenic and likely pathogenic variants were considered diagnostic. Variants of uncertain significance were evaluated in the context of the patient’s phenotype, inheritance pattern, in silico prediction tools, and available published evidence. When a VUS was identified in trans with a pathogenic variant in genes associated with autosomal recessive disorders and demonstrated strong genotype–phenotype concordance, its potential clinical relevance was considered during multidisciplinary case discussion; however, classification remained consistent with ACMG/AMP recommendations.

Molecular diagnoses were established only when the identified variants adequately explained the patient’s clinical phenotype according to the expected inheritance pattern and current evidence supporting disease causality.

### 2.5. Outcome Measures

The primary outcome of the study was the molecular diagnostic yield, defined as the proportion of patients in whom the ophthalmology-focused multigene panel established a definitive molecular diagnosis consistent with the clinical phenotype.

Secondary outcomes included characterization of the molecular spectrum of genetic eye diseases identified within the cohort, distribution of disease-causing genes, ACMG/AMP variant classification, inheritance patterns, zygosity, frequency of heterozygous carriers of autosomal recessive disorders, and characterization of unresolved cases. These outcome measures were selected to evaluate not only the diagnostic performance of the multigene panel but also its clinical applicability in a heterogeneous population representative of routine ophthalmic genetics practice.

### 2.6. Statistical Analysis

Descriptive statistical analyses were performed using IBM SPSS Statistics version 25 (IBM Corp., Armonk, NY, USA). Continuous variables were summarized as means, medians, ranges, and standard deviations where appropriate, whereas categorical variables were reported as frequencies and percentages.

The distribution of molecular diagnoses, disease-associated genes, inheritance patterns, zygosity, and variant classifications was analyzed descriptively. Age at clinical evaluation and age at symptom onset were summarized to characterize the study population. Given the observational nature of the study, no inferential statistical analyses were performed.

## 3. Results

### 3.1. Study Population

A total of 101 consecutive patients with suspected genetic eye diseases were included between January 2020 and August 2023. All patients completed the institutional diagnostic workflow, consisting of ophthalmologic evaluation followed by molecular analysis using an ophthalmology-focused multigene panel.

The cohort comprised 58 males (57.4%) and 43 females (42.6%). Age at evaluation ranged from birth to 88 years, with a mean of 36.5 years and a median of 37 years. Adults (≥18 years) represented 54.5% (*n* = 55) of the study population, whereas pediatric patients accounted for 45.5% (*n* = 46).

The study population reflected the broad spectrum of patients evaluated in a tertiary ophthalmic genetics clinic rather than a single disease category. The most common referral diagnoses included inherited retinal dystrophies, bilateral retinal detachment with suspected collagen disorder, optic atrophy, nystagmus, and foveal hypoplasia (Table 1), illustrating the marked clinical heterogeneity of GEDs.

### 3.2. Molecular Diagnostic Yield

**Figure 1:**
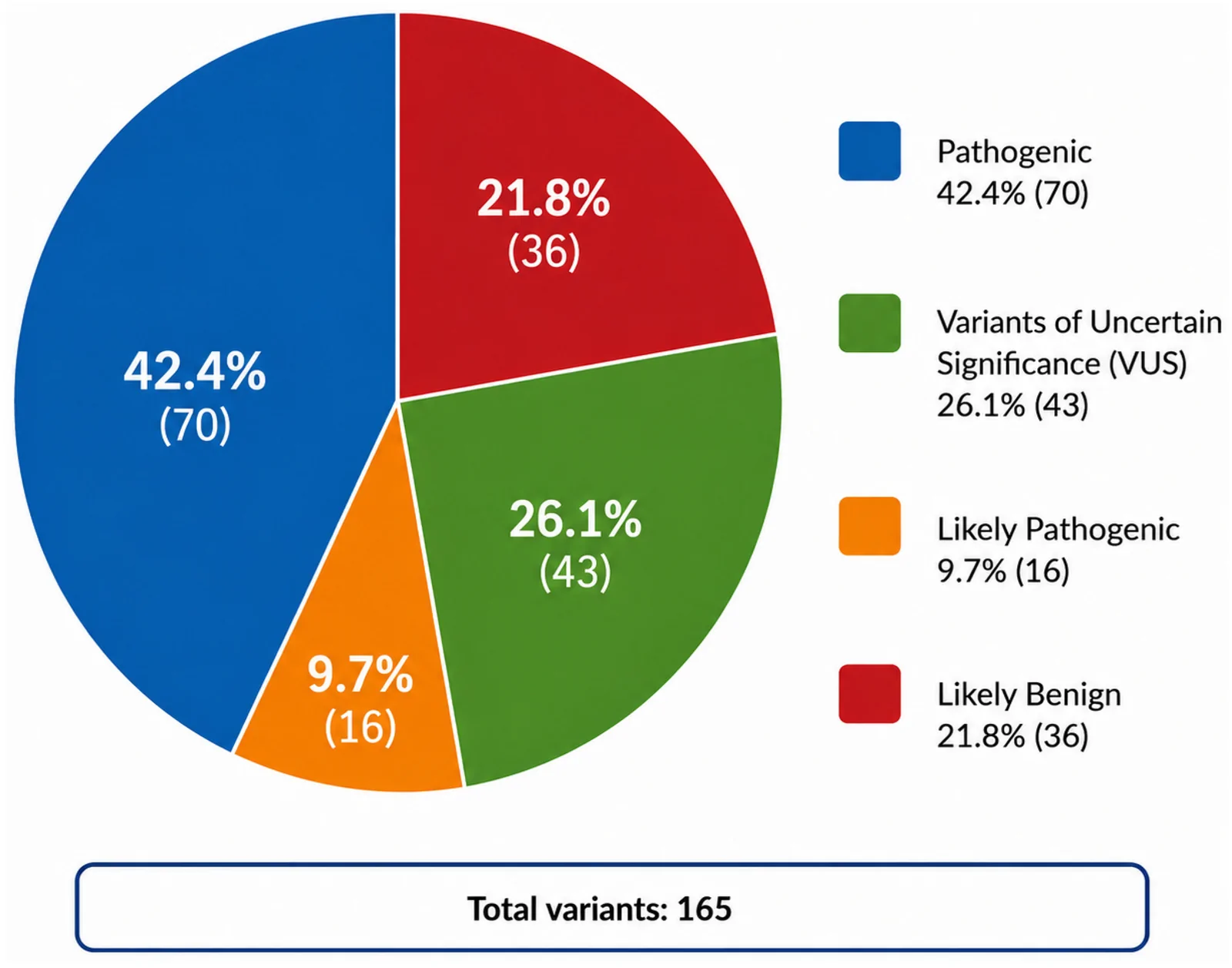
Classification of genetic variants identified in the study cohort according to the ACMG/AMP guidelines. A total of 165 variants were identified. Variants were classified as pathogenic (42.4%), likely pathogenic (9.7%), variants of uncertain significance (VUS) (26.1%), and likely benign (21.8%), following the American College of Medical Genetics and Genomics/Association for Molecular Pathology recommendations.

Molecular analysis identified a total of 165 sequence variants across the study cohort. According to ACMG/AMP criteria, 70 variants (42.4%) were classified as pathogenic, 16 (9.7%) as likely pathogenic, 43 (26.1%) as variants of uncertain significance (VUS), and 36 (21.8%) as likely benign (Figure 1).

Overall, the ophthalmology-focused multigene panel established a clinically actionable molecular diagnosis in 45 of 101 patients, corresponding to an overall molecular diagnostic yield of 44.6%. In addition, 18 individuals (17.8%) were identified as heterozygous carriers of pathogenic variants associated with autosomal recessive disorders, providing clinically relevant information for reproductive counseling despite the absence of a definitive molecular diagnosis.

### 3.3. Molecular Spectrum of Genetic Eye Diseases

The molecular diagnoses identified in this cohort encompassed a wide spectrum of genetic eye diseases. Usher syndrome was the most frequent molecular diagnosis, accounting for 22.2% (10/45) of molecularly confirmed cases, comprising nine patients with *USH2A*–associated Usher syndrome type 2 (20.0%) and one patient with *MYO7A*–associated Usher syndrome type 1B (2.2%). Stickler syndrome accounted for 17.8% (8/45) of confirmed diagnoses, including five patients with *COL2A1*–associated Stickler syndrome type 1 (11.1%) and three with *COL11A1*–associated Stickler syndrome type 2 (6.7%). Oculocutaneous albinism represented 6.7% (3/45) of confirmed diagnoses, including two patients with *OCA2*–associated oculocutaneous albinism type 2 (4.4%) and one with *TYR*–associated oculocutaneous albinism type 1 (2.2%). Alström syndrome was identified in two patients (4.4%) with pathogenic variants in *ALMS1*. Detailed patient-level genotype and associated phenotype information, including transcript, detected variant(s), zygosity, phase, inheritance pattern, and molecular diagnosis, is provided in Supplementary Table S2.

Less frequently identified conditions included inherited optic neuropathies, cone–rod dystrophy, Leber congenital amaurosis, vitreoretinopathies, anterior segment dysgenesis, Wolfram syndrome, X-linked retinoschisis, Traboulsi syndrome, and other rare hereditary ophthalmic disorders, illustrating the broad diagnostic scope achieved by the ophthalmology-focused multigene panel (Table 2).

### 3.4. Variant Spectrum, Inheritance Patterns, and Zygosity

Autosomal recessive disorders represented the predominant inheritance pattern, accounting for 68.9% (31/45) of all molecular diagnoses, followed by autosomal dominant disorders (28.9%, 13/45) and X-linked disease (2.2%, 1/45).

Regarding zygosity, compound heterozygous variants confirmed in trans were identified in 48.9% (22/45) of molecularly diagnosed patients. Heterozygous pathogenic variants accounted for 28.9% (13/45), homozygous pathogenic variants for 20.0% (9/45), and one individual (2.2%) had a hemizygous pathogenic variant associated with X-linked inheritance. Detailed patient-level variant information is provided in Supplementary Table S2.

Analysis of variant type demonstrated that missense variants were the most common molecular consequence, representing approximately half of all disease-causing variants, followed by nonsense variants, splice-site alterations, and small insertions/deletions.

### 3.5. Unresolved Cases

Despite comprehensive analysis using the ophthalmology-focused multigene panel, 18 patients (17.8%) remained without a definitive molecular diagnosis. Most unresolved cases presented clinical phenotypes compatible with inherited retinal disorders, including retinitis pigmentosa, Usher syndrome, Bardet–Biedl syndrome, cone–rod dystrophy, optic atrophy, and oculocutaneous albinism.

Several unresolved patients harbored variants of uncertain significance or single heterozygous pathogenic variants in genes associated with autosomal recessive disorders, precluding definitive molecular confirmation according to current ACMG/AMP criteria. These findings highlight the inherent limitations of current knowledge regarding variant interpretation rather than analytical performance of the sequencing assay.

Although additional approaches such as whole-genome sequencing, transcriptome analysis, long-read sequencing, or periodic variant reinterpretation may ultimately resolve a proportion of these cases, the present results show that ophthalmology-focused multigene panels establish a clinically actionable diagnosis in a substantial proportion of patients with suspected genetic eye diseases encountered in routine practice.

## 4. Discussion

In this study, an ophthalmology-focused multigene panel achieved a clinically actionable molecular diagnostic yield of 44.6% in a heterogeneous cohort of patients with suspected genetic eye diseases evaluated under real-world clinical conditions. More importantly, our findings support the use of comprehensive multigene panels as an effective first-line diagnostic strategy, particularly in healthcare systems where access to advanced ophthalmic phenotyping is limited [5,6,7,8,9,10,11,12,13,14,15].

The molecular diagnostic yield observed in our cohort is consistent with findings from international studies using targeted next-generation sequencing for hereditary ophthalmic disorders. Reported diagnostic yields range from approximately 40% to 70%, depending on patient selection, phenotype definition, sequencing strategy, and variant interpretation criteria (Table 3) [9,10,11,12,13,14,15,16,17,18]. Previously published Mexican cohorts have reported somewhat higher diagnostic rates, largely reflecting more phenotypically selected populations because most focused on highly selected patients with inherited retinal dystrophies after extensive pre-test phenotyping. In contrast, our cohort intentionally included consecutive patients with a broad range of suspected GEDs, including retinal dystrophies, inherited optic neuropathies, vitreoretinal disorders, developmental ocular anomalies, anterior segment disease, albinism, and syndromic conditions. Our findings therefore represent the performance achievable in routine tertiary clinical practice rather than in a narrowly selected disease-specific cohort.

Despite differences in cohort composition, the molecular spectrum identified in our study paralleled previous Mexican and international reports. *USH2A* was the most frequently implicated individual gene, accounting for 9 of 45 molecularly confirmed cases (20.0%), consistent with its established contribution to inherited retinal disease across diverse populations [9,10,11,12,13,14,15,16,17,18]. The predominance of autosomal recessive inheritance and the identification of Stickler syndrome and oculocutaneous albinism were also concordant with prior observations. These findings reinforce the reproducibility and clinical utility of ophthalmology-focused multigene panels across populations and healthcare systems.

A principal strength of this study is the incorporation of molecular testing into a real-world diagnostic workflow. Patients were referred according to clinical suspicion of a GED rather than after exhaustive electrophysiological characterization. Detailed phenotyping—including OCT, FAF, visual fields, and electrophysiological testing—remains essential for disease classification, functional assessment, prognosis, and genotype–phenotype correlation [19,20,21]. Nevertheless, these investigations require specialized infrastructure and expertise that are not universally available.

In Mexico and other middle-income settings, standardized electrophysiological testing may be unavailable at some referral centers or difficult for patients to access because of cost, travel, waiting times, and limited pediatric expertise. Diagnostic algorithms that require complete deep phenotyping before molecular testing may therefore prolong the diagnostic odyssey, delay genetic counseling and multidisciplinary evaluation, and postpone recognition of candidates for gene-specific therapies. Our findings suggest that early implementation of an ophthalmology-focused multigene panel may facilitate earlier molecular diagnosis while achieving a clinically meaningful diagnostic yield.

Based on these findings, we propose a pragmatic workflow in which a comprehensive ophthalmologic examination identifies patients with a high clinical suspicion of a GED, followed by early molecular testing with an ophthalmology-focused multigene panel. Once a molecular diagnosis is established—or when the result remains uncertain—targeted electrophysiological and imaging studies can be selected to refine phenotypic characterization, prognosis, disease monitoring, and genotype–phenotype correlation. This approach does not diminish the importance of deep phenotyping; rather, it uses molecular findings to direct subsequent investigations and optimize limited healthcare resources.

A similar shift has occurred in inherited neuromuscular disorders. Historically, diagnosis relied heavily on electrophysiological studies and muscle biopsy before molecular confirmation. The implementation of NGS has moved comprehensive genetic testing earlier in the diagnostic pathway, while invasive or highly specialized investigations are increasingly reserved for unresolved cases or for clarification of disease mechanisms and therapeutic planning [22,23,24]. Ophthalmic genetics may be undergoing a comparable transition in which molecular diagnosis increasingly guides, rather than invariably follows, advanced phenotypic investigations.

The expanding landscape of precision therapies further increases the clinical value of an early molecular diagnosis. Approval of gene replacement therapy for RPE65-associated retinal dystrophy and continued development of RNA-based, genome-editing, and mutation-specific therapies demonstrate the importance of identifying the causal gene and variant [27,28,29,30]. Molecular diagnosis also supports genetic counseling, cascade testing, multidisciplinary surveillance for syndromic manifestations, enrollment in natural history studies, and referral to gene-specific clinical trials.

This study has limitations. Its retrospective design limited the uniformity and completeness of phenotypic characterization, precluding detailed genotype–phenotype correlations for every disorder. The study was conducted at a single tertiary referral center and may not fully represent the spectrum of GEDs in other populations. In addition, the analysis did not compare alternative sequencing strategies or formally assess cost-effectiveness. Nevertheless, these limitations reflect routine clinical practice in many middle-income settings. The inclusion of consecutive referrals provides a realistic estimate of panel performance in a heterogeneous ophthalmic genetics population.

Collectively, our findings support earlier incorporation of ophthalmology-focused multigene panels into the evaluation of patients with suspected genetic eye diseases. In healthcare systems with limited access to specialized imaging or electrophysiological testing, early molecular testing may shorten the diagnostic odyssey, improve resource allocation, facilitate precision diagnosis and genetic counseling, and enable timely identification of candidates for gene-specific therapies. Advanced functional and imaging studies remain indispensable but can be deployed more selectively after molecular testing to refine prognosis, monitor progression, and strengthen genotype–phenotype correlations. Prospective multicenter studies incorporating standardized phenotyping, health-economic analysis, and longitudinal follow-up are needed to validate this workflow across different healthcare systems.

## 5. Conclusions

An ophthalmology-focused multigene panel established a molecular diagnosis in 44.6% of a heterogeneous, real-world cohort of patients with suspected genetic eye diseases. These findings support early panel testing as a practical first-line diagnostic strategy, particularly in healthcare systems where access to advanced ophthalmic phenotyping is limited. Molecular testing should complement rather than replace detailed ophthalmologic evaluation, enabling subsequent imaging and electrophysiological studies to be selected in a more targeted manner. Earlier molecular diagnosis can facilitate genetic counseling, syndromic surveillance, cascade testing, prognostic assessment, and access to emerging gene-specific therapies.

## Figures and Tables

**Table 1:** Demographic and Clinical Characteristics of the Study Cohort.

Characteristic	Value
Total patients	101
Male, *n* (%)	58 (57.4)
Female, *n* (%)	43 (42.6)
Mean age, years	36.5
Median age, years	37
Age range	Newborn–88 years
Adults (≥18 years), *n* (%)	55 (54.5)
Pediatric patients (<18 years), *n* (%)	46 (45.5)
Most frequent referral diagnosis	Inherited retinal disorder
Other common referral diagnoses	Bilateral retinal detachment, optic nerve atrophy, nystagmus, foveal hypoplasia

Baseline demographic and clinical characteristics of patients with suspected genetic eye diseases. Data are presented as number (%) unless otherwise indicated. Patients were consecutively referred from the Department of Ophthalmology to the Department of Medical Genetics for molecular evaluation.

**Table 2:** Molecular Spectrum of Genetic Eye Diseases Identified in the Cohort.

Molecular Diagnosis	Major Gene(s)	Patients (*n*)	% of Confirmed Diagnoses	Inheritance
Usher syndrome	*USH2A*, *MYO7A*	10	22.2	AR
Stickler syndrome	*COL2A1*, *COL11A1*	8	17.8	AD
Oculocutaneous albinism	*OCA2*, *TYR*	3	6.7	AR
Alström syndrome	*ALMS1*	2	4.4	AR
Other genetic eye diseases	*ABCA4*, *ACO2*, *ASPH*, *BBS7*, *BEST1*, *CERKL*, *CNGB1*, *COL18A1*, *CPAMD8*, *LRP5*, *NDUFS4*, *NMNAT1*, *OPA1*, *OTX2*, *PROM1*, *PRPH2*, *RPE65*, *RS1*, *TTLL5*, *WFS1*	22	48.9	AR/AD/XL
Total	—	45	100	—

Molecular diagnoses are presented as mutually exclusive categories among the 45 patients with a confirmed molecular diagnosis. The Usher syndrome group includes nine patients with *USH2A*-associated Usher syndrome type 2 and one patient with *MYO7A*-associated Usher syndrome type 1B. Stickler syndrome includes five *COL2A1*-associated type 1 cases and three *COL11A1*-associated type 2 cases. Oculocutaneous albinism includes two *OCA2*-associated type 2 cases and one *TYR*-associated type 1 case. The “other genetic eye diseases” category comprises individually uncommon molecular diagnoses. AD, autosomal dominant; AR, autosomal recessive; XL, X-linked. Detailed patient-level genotype–phenotype data are provided in Supplementary Table S2.

**Table 3:** Comparison of Molecular Diagnostic Studies Using Next-Generation Sequencing in Genetic Eye Diseases.

Study	Country	Cohort (*n*)	Diagnostic Yield	Molecular Approach	Main Clinical Contribution
Jespersgaard et al., 2019) [9]	Denmark	677	48.0%	Targeted NGS panel	Large national retinal dystrophy cohort
Colombo et al., 2021 [10]	Italy	591	55.2%	Targeted NGS	Nonsyndromic RP and Usher syndrome
Liu et al., 2021 [12]	China	800 families	60.1%	Comprehensive genetic testing	Large nonsyndromic IRD cohort
Patel et al., 2019 [15]	United Kingdom	1234	51.0%	Oculome panel	Broad developmental and genetic eye disorders
Zenteno et al., 2020 [16]	Mexico	143	66.0%	Targeted NGS panel	Mexican IRD molecular spectrum
Villanueva-Mendoza et al., 2021 [17]	Mexico	144	72.9%	364-gene panel	Expanded Mexican IRD landscape
Villafuerte-de la Cruz et al., 2024 [18]	Mexico	126	74.6%	293-gene panel	Northeastern Mexican IRD cohort
Present study	Mexico	101	44.6%	Ophthalmology-focused multigene panel	Real-world heterogeneous GED cohort

Diagnostic yield was defined according to each publication and may not be directly comparable because of differences in patient selection, phenotype composition, sequencing strategy, bioinformatic pipelines, and variant interpretation. GED, genetic eye disease; IRD, inherited retinal dystrophy; NGS, next-generation sequencing; RP, retinitis pigmentosa. The present study evaluated ophthalmology-focused panel testing across a broad, heterogeneous spectrum of suspected genetic eye diseases in routine tertiary practice.

## Data Availability

The data supporting the findings of this study are available from the corresponding author upon reasonable request. The data are not publicly available because they contain clinical and genetic information that could compromise participant privacy.

## Supplementary Materials

The following supporting information can be downloaded at: https://www.mdpi.com/article/10.3390/genes17091092/s1. Supplementary Table S1: Complete List of Genes Included in the Ophthalmology-Focused Multigene Panel; Table S2: Molecular Diagnoses in the 45 Cases.

## References

[1] Hamel C. (2006). Retinitis pigmentosa. Orphanet J. Rare Dis..

[2] Verbakel S.K., van Huet R.A.C., Boon C.J.F., den Hollander A.I., Collin R.W.J., Klaver C.C.W., Hoyng C.B., Roepman R., Klevering B.J. (2018). Non-syndromic retinitis pigmentosa. Prog. Retin. Eye Res..

[3] Hanany M., Rivolta C., Sharon D. (2020). Worldwide carrier frequency and genetic prevalence of autosomal recessive inherited retinal diseases. Proc. Natl. Acad. Sci. USA.

[4] Duncan J.L., Pierce E.A., Laster A.M., Daiger S.P., Birch D.G., Ash J.D., Iannaccone A., Flannery J.G., Sahel J.A., Zack D.J. (2018). Inherited retinal degenerations: Current landscape and knowledge gaps. Transl. Vis. Sci. Technol..

[5] Dockery A., Whelan L., Humphries P., Farrar G.J. (2021). Next-generation sequencing applications for inherited retinal diseases. Int. J. Mol. Sci..

[6] Nash B.M., Wright D.C., Grigg J.R., Bennetts B., Jamieson R.V. (2015). Retinal dystrophies, genomic applications in diagnosis and prospects for therapy. Transl. Pediatr..

[7] Wright C.F., FitzPatrick D.R., Firth H.V. (2018). Paediatric genomics: Diagnosing rare disease in children. Nat. Rev. Genet..

[8] Broadgate S., Yu J., Downes S.M., Halford S. (2017). Unravelling the genetics of inherited retinal dystrophies: Past, present and future. Prog. Retin. Eye Res..

[9] Jespersgaard C., Fang M., Bertelsen M., Dang X., Jensen H., Chen Y., Bech N., Dai L., Rosenberg T., Zhang J. (2019). Molecular genetic analysis using targeted NGS analysis of 677 individuals with retinal dystrophy. Sci. Rep..

[10] Colombo L., Maltese P.E., Castori M., El Shamieh S., Zeitz C., Audo I., Zulian A., Marinelli C., Benedetti S., Costantini A. (2021). Molecular epidemiology in 591 Italian probands with nonsyndromic retinitis pigmentosa and Usher syndrome. Investig. Ophthalmol. Vis. Sci..

[11] Britten-Jones A.C., Gocuk S.A., Goh K.L., Huq A., Edwards T.L., Ayton L.N. (2023). The diagnostic yield of next-generation sequencing in inherited retinal diseases: A systematic review and meta-analysis. Am. J. Ophthalmol..

[12] Liu X., Tao T., Zhao L., Li G., Yang L. (2021). Molecular diagnosis based on comprehensive genetic testing in 800 Chinese families with non-syndromic inherited retinal dystrophies. Clin. Exp. Ophthalmol..

[13] Chen X., Liu X., Sheng X., Gao X., Zhang X., Li Z., Li H., Liu Y., Rong W., Zhao K. (2015). Targeted next-generation sequencing reveals novel EYS mutations in Chinese families with autosomal recessive retinitis pigmentosa. Sci. Rep..

[14] Kang S., Lam B.L., Lee W., Berrocal A.M., Gregori N.Z., Mendoza-Santiesteban C.E., Sengillo J.D. (2026). Genetic testing in inherited retinal disease: Current strategies and future directions. J. Pers. Med..

[15] Patel A., Hayward J.D., Tailor V., Nyanhete R., Ahlfors H., Gabriel C., Jannini T.B., Abbou-Rayyah Y., Henderson R., Nischal K.K. (2019). The Oculome Panel Test: Next-generation sequencing to diagnose a diverse range of genetic developmental eye disorders. Ophthalmology.

[16] Zenteno J.C., García-Montaño L.A., Cruz-Aguilar M., Ronquillo J., Rodas-Serrano A., Aguilar-Castul L., Matsui R., Vencedor-Meraz C.I., Chacón-Camacho O.F. (2020). Extensive genic and allelic heterogeneity underlying inherited retinal dystrophies in Mexican patients molecularly analyzed by next-generation sequencing. Mol. Genet. Genom. Med..

[17] Villanueva-Mendoza C., Tuson M., Apam-Garduño D., de Castro-Miró M., Tonda R., Trotta J.R., Marfany G., Valero R., Cortés-González V., Gonzàlez-Duarte R. (2021). The genetic landscape of inherited retinal diseases in a Mexican cohort: Genes, mutations and phenotypes. Genes.

[18] Villafuerte-de la Cruz R.A., Garza-Garza L.A., Garza-Leon M., la Torre C.R.-D., Parra-Bernal C., Vazquez-Camas I., Ramos-Gonzalez D., Rangel-Padilla A., Barros-Palau A.E., Nava-García J. (2024). Spectrum of variants associated with inherited retinal dystrophies in Northeast Mexico. BMC Ophthalmol..

[19] Cornish E.E., Vaze A., Jamieson R.V., Grigg J.R. (2021). The electroretinogram in the genomics era: Outer retinal disorders. Eye.

[20] Sodi A., Banfi S., Testa F., Della Corte M., Passerini I., Pelo E., Rossi S., Simonelli F., Italian IRD Working Group (2021). RPE65-associated inherited retinal diseases: Consensus recommendations for eligibility to gene therapy. Orphanet J. Rare Dis..

[21] Robson A.G., Frishman L.J., Grigg J., Hamilton R., Jeffrey B.G., Kondo M., Li S., McCulloch D.L. (2022). ISCEV Standard for full-field clinical electroretinography (2022 update). Doc. Ophthalmol..

[22] Bönnemann C.G., Wang C.H., Quijano-Roy S., Deconinck N., Bertini E., Ferreiro A., Muntoni F., Sewry C., Béroud C., Mathews K.D. (2014). Diagnostic approach to the congenital muscular dystrophies. Neuromuscul. Disord..

[23] Monies D., Alhindi H.N., Almuhaizea M.A., Abouelhoda M., Alazami A.M., Goljan E., Alyounes B., Alshaibani K., Alruwaili J., Jaafar A. (2016). A first-line diagnostic assay for limb-girdle muscular dystrophy and other myopathies. Hum. Genom..

[24] Straub V., Clause A.R., Donkervoort S., Kang P.B., Laverty C.G., Niu Z., Wicklund M.P., Bönnemann C.G., Cooper S.T., Díaz-Manera J. (2025). Expert consensus on genetic diagnostic approaches for patients with limb-girdle muscular dystrophy. Neurology.

[25] Riggs E.R., Andersen E.F., Cherry A.M., Kantarci S., Kearney H., Patel A., Raca G., Ritter D.I., South S.T., Thorland E.C. (2020). Technical standards for the interpretation and reporting of constitutional copy-number variants: A joint consensus recommendation of the ACMG and ClinGen. Genet. Med..

[26] Richards S., Aziz N., Bale S., Bick D., Das S., Gastier-Foster J., Grody W.W., Hegde M., Lyon E., Spector E. (2015). Standards and guidelines for the interpretation of sequence variants: A joint consensus recommendation of the American College of Medical Genetics and Genomics and the Association for Molecular Pathology. Genet. Med..

[27] Russell S., Bennett J., Wellman J.A., Chung D.C., Yu Z.F., Tillman A., Wittes J., Pappas J., Elci O., McCague S. (2017). Efficacy and safety of voretigene neparvovec in patients with RPE65-mediated inherited retinal dystrophy: A randomized, controlled, open-label, phase 3 trial. Lancet.

[28] Pierce E.A., Bennett J. (2015). The status of RPE65 gene therapy trials: Safety and efficacy. Cold Spring Harb. Perspect. Med..

[29] Ellingford J.M., Barton S., Bhaskar S., O’Sullivan J., Williams S.G., Lamb J.A., Panda B., Sergouniotis P.I., Gillespie R.L., Daiger S.P. (2016). Whole genome sequencing increases molecular diagnostic yield compared with current diagnostic testing for inherited retinal disease. Ophthalmology.

[30] den Hollander A.I., Black A., Bennett J., Cremers F.P.M. (2010). Lighting a candle in the dark: Advances in genetics and gene therapy of recessive retinal dystrophies. J. Clin. Investig..

