# Supplementary material for: Early Molecular Testing for Presumptive Genetic Eye Diseases
Source: Genes (Basel). 2026 Sep 10;17(9):1092. doi: 10.3390/genes17091092 (PMC13606511; doi:10.3390/genes17091092)
Supplement: Supplementary file 1 [file genes-17-01092-s001.zip › genes-4477634-supplementary.pdf]

Supplementary material

Table S1. Complete List of Genes Included in the Ophthalmology-Focused Multigene Panel.

ABCA4	*ABCC6*	*ABHD12*	*ACBD5*	*ACO2*	*ADAM9*
ADAMTS18	*ADAMTSL4*	*ADGRA3*	*ADGRV1*	*ADIPOR1*	*AGBL5*
AHI1	*AHR*	*AIPL1*	*ALMS1*	*ARHGEF18*	*ARL13B*
ARL2BP	*ARL3*	*ARL6*	*ARMC9*	*ARSG*	*ASRGL1*
ATF6	*ATOH7*	*B9D1*	*BBIP1*	*BBS1*	*BBS10*
BBS12	*BBS2*	*BBS4*	*BBS5*	*BBS7*	*BBS9*
BEST1	*C10orf11*	*C12orf65*	*C1QTNF5*	*C8orf37*	*CA4*
CABP4	*CACNA1F*	*CACNA2D4*	*CAPN5*	*CC2D2A*	*CCT2*
CDH23	*CDH3*	*CDHR1*	*CEP164*	*CEP19*	*CEP250*
CEP290	*CEP41*	*CEP78*	*CEP83*	*CERKL*	*CFAP410*
CHM	*CIB2*	*CISD2*	*CLCC1*	*CLN2(TPP1)*	*CLN3*
CLN5	*CLN6*	*CLN8*	*CLRN1*	*CLUAP1*	*CNGA1*
CNGA3	*CNGB1*	*CNGB3*	*CNNM4*	*COL11A1*	*COL11A2*
COL18A1	*COL2A1*	*COL9A1*	*COL9A2*	*COL9A3*	*CPLANE1*
CRB1	*CRX*	*CSPP1*	*CTNNA1*	*CTSD*	*CWC27*
CYP4V2	*DHDDS*	*DHX32*	*DHX38*	*DNAJC17*	*DRAM2*
DSCAML1	*DTHD1*	*EFEMP1*	*ELOVL4*	*EMC1*	*ERCC6*
EXOSC2	*EYS*	*FAM161A*	*FBLN5*	*FLVCR1*	*FRMD7*
FSCN2	*FZD4*	*GDF6*	*GNAT1*	*GNAT2*	*GNB3*
GNPTG	*GNS*	*GPR143*	*GPR179*	*GPR45*	*GRM6*
GRN	*GUCA1A*	*GUCA1B*	*GUCY2D*	*HARS*	*HGSNAT*
HK1	*HMCN1*	*HMX1*	*IDH3A*	*IDH3B*	*IFT140*
IFT172	*IFT27*	*IFT43*	*IFT74*	*IFT80*	*IFT81*
IFT88	*IMPDH1*	*IMPG1*	*IMPG2*	*INPP5E*	*INVS*
IQCB1	*ITM2B*	*JAG1*	*KCNJ13*	*KCNV2*	*KIAA0586*
KIAA1549	*KIF11*	*KIF7*	*KIZ*	*KLHL7*	*LCA5*
LRAT	*LRIT3*	*LRP2*	*LRP5*	*LYST*	*LZTFL1*
MAK	*MAPKAPK3*	*MERTK*	*MFN2*	*MFRP*	*MFSD8*
MIR204	*MKKS*	*MKS1*	*MPDZ*	*MTPAP*	*MTTP*
MYO7A	*NAGLU*	*NBAS*	*NDP*	*NEK2*	*NEUROD1*
NMNAT1	*NPHP1*	*NPHP3*	*NPHP4*	*NR2E3*	*NR2F1*
NRL	*NYX*	*OAT*	*OCA2*	*OFD1*	*OPA1*
OPA3	*OPN1SW*	*OR2W3*	*OTX2*	*P3H2*	*PAX2*
PAX6	*PCARE*	*PCDH15*	*PCYT1A*	*PDE6A*	*PDE6B*
PDE6C	*PDE6D*	*PDE6G*	*PDE6H*	*PDZD7*	*PEX1*
PEX10	*PEX11B*	*PEX12*	*PEX13*	*PEX14*	*PEX16*
PEX19	*PEX2*	*PEX26*	*PEX3*	*PEX5*	*PEX6*
PEX7	*PHYH*	*PITPNM3*	*PLA2G5*	*PLK4*	*PNPLA6*
POC1B	*POC5*	*POMGNT1*	*PPT1*	*PRCD*	*PRDM13*
PROM1	*PRPF3*	*PRPF31*	*PRPF4*	*PRPF6*	*PRPF8*
PRPH2	*PRPS1*	*RAB28*	*RAX2*	*RBP1*	*RBP3*
RBP4	*RCBTB1*	*RD3*	*RDH11*	*RDH12*	*RDH5*
REEP6	*RGR*	*RGS9*	*RGS9BP*	*RHO*	*RIMS1*
RLBP1	*ROM1*	*RP1*	*RP1L1*	*RP2*	*RP9*
RPE65	*RPGR*	*RPGR(ORF15)*	*RPGRIP1*	*RPGRIP1L*	*RS1*
RTN4IP1	*SAG*	*SAMD11*	*SCLT1*	*SDCCAG8*	*SEMA4A*
SGSH	*SIX6*	*SLC24A1*	*SLC24A5*	*SLC45A2*	*SLC7A14*
SNRNP200	*SPATA7*	*SPP2*	*TCTN1*	*TCTN2*	*TCTN3*

TEAD1	*TIMM8A*	*TIMP3*	*TMED7*	*TMEM107*	*TMEM126A*
TMEM138	*TMEM216*	*TMEM231*	*TMEM237*	*TMEM67*	*TOPORS*
TRAF3IP1	*TREX1*	*TRIM32*	*TRNT1*	*TRPM1*	*TSPAN12*
TTC21B	*TTC8*	*TTLL5*	*TTPA*	*TUB*	*TUBGCP4*
TUBGCP6	*TULP1*	*TYR*	*TYRP1*	*UNC119*	*USH1C*
USH1G	*USH2A*	*VCAN*	*VPS13B*	*WDPCP*	*WDR19*
WDR34	*WFS1*	*WHRN*	*ZNF408*	*ZNF423*	*ZNF513*

The panel comprises genes associated with inherited retinal disorders, optic neuropathies, vitreoretinal diseases, anterior segment dysgenesis, ocular developmental disorders, albinism, ciliopathies, syndromic ophthalmic diseases, and other hereditary ocular conditions. Genes are presented in alphabetical order.

Table S2: Molecular Diagnoses in the 45 Cases.

Case	Gene	Transcript	Detected variant(s)	Zygosity	Inheritance	Molecular diagnosis
1	*ABCA4*	NM_000350.2	c.5318C>T, p.(Ala1773Val); c.6686T>C, p.(Leu2229Pro)	Compound heterozygous	AR	*ABCA4*-associated retinal dystrophy
2	*ABCA4*	NM_000350.2	c.5318C>T, p.(Ala1773Val); c.6686T>C, p.(Leu2229Pro)	Compound heterozygous	AR	*ABCA4*-associated retinal dystrophy
3	*ACO2*	NM_001098.2	c.2035C>T; c.1699C>T	Compound heterozygous	AR	Optic atrophy 9
4	*ALMS1*	NM_015120.4	c.4937C>A, p.(Ser1646*)	Homozygous	AR	Alström syndrome
5	*ALMS1*	NM_015120.4	c.2755del, p.(Ser919Profs*15) ; c.1730C>G, p.(Ser577*)	Compound heterozygous	AR	Alström syndrome
6	*ASPH*	NM_004318.4	Deletion exons 20–21; c.1892G>A, p.(Trp631*)	Compound heterozygous	AR	Traboulsi syndrome
7	*BBS7*	NM_176824.2	c.1334A>C, p.(Tyr445Ser); c.601+3A>G	Compound heterozygous	AR	Bardet–Biedl syndrome 7
8	*BEST1*	NM_004183.3	c.851A>G, p.(Tyr284Cys)	Heterozygous	AD	Vitreoretinochoroidopathy
9	*CERKL*	NM_001030311.2	c.898+2C>G, (Splice donor)	Homozygous	AR	Retinitis pigmentosa 26
10	*CNGB1*	NM_001297.4	c.3689del, p.(Gly1230Alafs*)	Homozygous	AR	Retinitis pigmentosa 45
11	*COL11A1*	NM_001854.3	c.4177G>T, p.(Gly1393Cys)	Heterozygous	AD	Stickler syndrome type 2
12	*COL11A1*	NM_001854.3	c.729_739del, p.(Cys243*)	Heterozygous	AD	Stickler syndrome type 2
13	COL11A1	NM_001854.3	c.4177G>T,p.(Gly1393Cys)	Heterozygous	AD	Stickler syndrome type 2
14	*COL2A1*	NM_001844.4	c.460G>T, p.(Glu154*)	Heterozygous	AD	Stickler syndrome type 1
15	*COL2A1*	NM_001844.4	c.1833+2dup, p?	Heterozygous	AD	Stickler syndrome type 1
16	*COL2A1*	NM_001844.4	c.85C>T, p.(Gln29*)	Heterozygous	AD	Stickler syndrome type 1
17	*COL2A1*	NM_001844.4	c.460G>T, p.(Glu154*)	Heterozygous	AD	Stickler syndrome type 1
18	*COL2A1*	NM_001844.4	c.460G>T, p.(Glu154*)	Heterozygous	AD	Stickler syndrome type 1
19	*COL18A1*	NM_130445.3	c.2518_2521dup, p.(Pro841Argfs*24); c.3527_3540delinsAGTG, p.(Met1176Lysfs*69)	Compound heterozygous	AR	Knobloch syndrome type 1
20	*CPAMD8*	NM_015692.2	c.3730T>A; c.1899+1_1899+4del (splice site)	Compound heterozygous	AR	Anterior segment dysgenesis 8
21	*LRP5*	NM_002335.3	c.3553G>A, p.(Gly1185Arg); c.685C>T, p.(Arg229Trp)	Compound heterozygous	AR	Exudative vitreoretinopathy 4
22	*MYO7A*	NM_000260.3	c.3G>A, p.(Met1?)	Homozygous	AR	Usher syndrome type 1
23	*NDUFS4*	NM_002495.4	c.497G>A p.(Trp166Ter)	Homozygous	AR	Leber optic neuropathy
24	*NMNAT1*	NM_022787.3	c.589A>G, (p.Ile197Val) ;c.769G>A p.(Glu629Aspfs*23)	Compound heterozygous	AR	Leber congenital amaurosis 9
25	*OCA2*	NM_000275.2	c.1000G>A, p.(Ala334Thr)	Homozygous	AR	Oculocutaneous albinism type 2
26	*OCA2*	NM_000275.2	c.632C>T, p.(Pro211Leu)	Homozygous	AR	Oculocutaneous albinism type 2
27	*OPA1*	NM_015560.2	c.89T>C (p.Leu30Pro)	Heterozygous	AD	Optic atrophy 1
28	*OPA1*	NM_015560.2	Deletion exons 1–14	Heterozygous	AD	Optic atrophy 1
29	*OTX2*	NM_172337.2	Deletion (entire coding sequence)	Heterozygous	AD	*OTX2*-related developmental eye disorder
30	*PROM1*	NM_006017.2	c.2130+2del, (splice site); c.1354dup, p.(Tyr452Leufs*13)	Compound heterozygous	AR	*PROM1* associated retinal dystrophy
31	*PRPH2*	NM_000322.4	c.514C>T p.(Arg172Trp)	Heterozygous	AD	Leber congenital amaurosis 18

32	*RPE65*	NM_000329.2	c.432C>T (p.Tyr144*); c.370C>T, (p.Arg124*)	Compound heterozygous	AR	*RPE65*-associated retinal dystrophy
33	*RS1*	NM_000330.3	c.208G>A, p.(Gly70Ser)	Hemizygous	XL	X-linked retinoschisis
34	*TTLL5*	NM_015072.4	c.3552del, p.(Ser1185Profs*6); c.2388-1G>C, p.?	Compound heterozygous	AR	Cone–rod dystrophy 19
35	*TYR*	NM_000372.4	c.132T>A, p.(Ser44Arg)	Homozygous	AR	Oculocutaneous albinism type 1
36	*USH2A*	NM_206933.2	c.4957C>T, p.(Arg1653*); c.11387C>T, p.(Pro3796Leu)	Compound heterozygous	AR	Usher syndrome type 2
37	*USH2A*	NM_206933.2	c.12575G>A, p.(Arg4192His); c.5941A>G, p.(Arg1981Gly)	Compound heterozygous	AR	Usher syndrome type 2
38	*USH2A*	NM_206933.2	c.12067-2A>G (splice acceptor); c.2299del, p.(Glu767Serfs*21)	Compound heterozygous	AR	Usher syndrome type 2
39	*USH2A*	NM_206933.2	c.2299del, p.(Glu767Serfs*21); c.12067-2A>G (splice acceptor)	Compound heterozygous	AR	Usher syndrome type 2
40	*USH2A*	NM_206933.2	c.2299del, p.(Glu767Serfs*21); c.12067-2A>G (splice acceptor)	Compound heterozygous	AR	Usher syndrome type 2
41	*USH2A*	NM_206933.2	c.12067-2A>G (splice acceptor); c.2299del, p.(Glu767Serfs*21)	Compound heterozygous	AR	Usher syndrome type 2
42	*USH2A*	NM_206933.2	c.11431_11434del, p.(Leu3811Metfs*5); c.5278del, p.(Asp1760Metfs*10)	Compound heterozygous	AR	Usher syndrome type 2
43	*USH2A*	NM_206933.2	c.2276G>T, p.(Cys759Phe); c.1551-1G>A (splice acceptor)	Compound heterozygous	AR	Usher syndrome type 2
44	*USH2A*	NM_206933.2	c.2299del, p.(Glu767Serfs*21)	Homozygous	AR	Usher syndrome type 2
45	*WFS1*	NM_006005.3	c.409_424dup, p.(Val142Glyfs*110); c.2189G>A, p.(Trp730*)	Compound heterozygous	AR	Wolfram-like syndrome 1

Supplementary Table S2. Patient-level molecular findings for the 45 individuals with a confirmed molecular diagnosis. For each patient, the implicated gene, reference transcript, detected variant(s), zygosity, inheritance pattern, and molecular diagnosis are provided. Variants are reported using HGVS nomenclature based on the indicated reference transcripts. For autosomal recessive conditions with two heterozygous variants, the variants were confirmed to be in trans. AD, autosomal dominant; AR, autosomal recessive; XL, X-linked.
